# Immunization Against Hepatitis A Virus and Hepatitis B Virus in Patients with Chronic Liver Disease: Are We Doing a Good Job?

**DOI:** 10.7759/cureus.2528

**Published:** 2018-04-24

**Authors:** Rutaba Tajammal, Ijlal Akbar Ali, Taseen Syed, Salman Nusrat

**Affiliations:** 1 Internal Medicine, University of Oklahoma Health Sciences Center, Oklahoma City, USA; 2 Section of Digestive Diseases and Nutrition, University of Oklahoma Health Sciences Center, Oklahoma City, USA; 3 Gasteroenterology, University of Oklahoma Health Sciences Center, Oklahoma City, USA

**Keywords:** hepatitis b, vaccination, hepatitis a, chronic liver disease, primary care, cirrhosis

## Abstract

Introduction: In the era of highly effective vaccines for Hepatitis A Virus (HAV) and Hepatitis B Virus (HBV), acute viral hepatitis in patients with a chronic liver disease remains a public health concern. Vaccination for HAV and HBV is endorsed by all liver society guidelines. The aim of our study was to determine the rates of immunization in an internal medicine resident clinic.

Methods: We identified patients with a chronic liver disease seen at the University of Oklahoma Internal Medicine resident clinic between June 2014 and May 2015. ICD-9 code 571 was used to identify patients with a chronic liver disease. Vaccination records and patient data were reviewed.

Results: A total of 141 patients with a chronic liver disease (mean age 54.1 years, 56% males) were identified. Almost half of the patients (47.5%) were also being seen in the gastroenterology clinic. During the internal medicine resident clinic visit, vaccination against HAV and HBV was addressed for 50% and 46% of the patients, respectively. Patients being seen by senior residents were more likely to be immunized against HAV (OR 2.7, p=0.009) and HBV (OR 2.1, p=0.03). Patients followed in the GI clinic were more likely to be immunized against HAV (OR 2.1, p= 0.02) and HBV (OR 2.0, p=0.02). The gender of the treating physician and etiology had no impact on vaccination rates.

Discussion: Immunization rates for HAV and HBV remain subpar despite clear guidelines for patients with a chronic liver disease. This provides an important avenue for improvement. Different strategies, including resident education, developing vaccination protocols, and referral to the gastroenterology clinic, are likely to improve vaccination status for patients with chronic liver diseases.

## Introduction

In the era of highly effective vaccines for Hepatitis A Virus (HAV) and Hepatitis B Virus (HBV), acute viral hepatitis remains a public health concern [1]. As compared to the general population, patients with chronic liver diseases are predisposed to severe hepatitis and possible liver failure if they contract an HAV or HBV infection [2-4]. As the prevalence of non-alcoholic fatty liver disease (NAFLD) and cirrhosis rises in the hospitalized patient population, patients with an acute hepatitis infection are likely to have a prolonged and complicated hospital course [2-4]. Patients with a pre-existing liver disease are also more likely to develop acute hepatitis-related complications, including encephalopathy and/or ascites [5-6]. Studies have shown that HAV and HBV vaccination is safe and effective in patients with chronic liver diseases [2]. The Centers for Disease Control and Prevention, the National Institutes of Health, the Veteran's Health Administration, and the American Liver Foundation are among the organizations endorsing vaccination of patients with chronic liver diseases [7].

The aim of our study was to determine the rates of HAV and HBV vaccination among patients with chronic liver diseases presenting to the internal medicine resident clinic at the University of Oklahoma Health Sciences Center.

## Materials and methods

Patients with chronic liver diseases seen at University of Oklahoma Internal Medicine resident clinic between June 2014 and May 2015 were identified using ICD-9 code 571. Demographic data, the etiology of the liver disease, clinic information, and treating physician information were extracted. Vaccination records were also reviewed. Patients were considered immunized if they had documented immunoglobulin G (IgG) HAV antibody (Ab) and IgG HBV surface antibody (HBV sAb) or they had documented vaccination administered in the clinic.

## Results

A total of 141 patients with a mean age of 54.1 years and 56% males were identified. Hepatitis C Virus (HCV) was the most common etiology (48%) followed by alcoholic liver disease in 21% and NAFLD in 20%. Almost half of the patients (47.5%) were also cared for in the gastroenterology clinic. During the internal medicine resident clinic visit, vaccination against HAV and HBV was addressed for 50% and 46% of the patients, respectively. IgG Ab against HAV was present in 47.5% patients, and 2.5% were identified as being immunized by two or more documented vaccinations against HAV. HBV sAb was present in 40%, and 6% were identified as being immunized by three documented vaccinations against HBV. Patients being seen by a senior medicine resident (PGY2 to PGY4) were more likely to be immunized against HAV (odds ratio (OR) 2.7, confidence interval (CI) 1.2-5.8, p=0.009) and HBV (OR 2.1, CI 1.05-4.4, p=0.03). Patients who were also being followed in the GI clinic were more likely to be immunized against HAV (OR 2.1, CI 1.3-3.9, p= 0.02) and HBV (OR 2.0, CI 1.1-3.7, p=0.02). The gender of the treating physician and the etiology of the chronic liver disease had no impact on documenting vaccination. The impact of different factors on vaccination rates for patients with chronic liver diseases is presented in Table 1.

**Table 1:** Impact of Different Factors on Vaccination Rates for Patients with Chronic Liver Diseases OR: odds ratio; CI: confidence interval

	HAV vaccination	HBV vaccination
Senior Residents (PGY2 to PGY4)	OR 2.7, CI 1.2-5.8, p=0.009	OR 2.1, CI 1.05-4.4, p=0.03
GI Clinic Follow-Up	OR 2.1, CI 1.3-3.9, p= 0.02	OR 2.0, CI 1.1-3.7, p=0.02
Etiology of Chronic Liver Disease (HCV)	OR 1.2, CI 0.7-1.4, p=0.7	OR 1.1, CI 0.7-1.3, p=0.8
Gender (Male)	OR 1.1, CI 0.5-1.3, p=0.6	OR 1.2, CI 0.4-1.9, p=0.8

## Discussion

Preventive care is the best and the most cost-effective way to improve patient outcomes and prevent untoward complications [8]. There are limited studies looking at the vaccination rates for patients with chronic liver diseases. Although vaccination rates for HAV and HBV in patients with chronic liver diseases have been variable, in most cases, they remain subpar [9]. One study showed that vaccination rates are lower in the primary care clinic as compared to the specialty clinic [10].

The resident clinic at a university hospital is an important avenue to provide quality care to patients with limited access to healthcare. Most patients coming to the resident clinic fall in the low-income category and are uninsured or underinsured [11]. This means that the resident clinic visit may be the only place for them to seek comprehensive healthcare. Most patients don’t have access to specialty care and, as seen in our study, less than half of the patients were being seen in the gastroenterology specialty clinic.

There can be multiple reasons for low vaccination rates in the resident clinic, including busy workload and lack of knowledge of current guidelines. Studies have highlighted variations in quantifiable outcomes and adherence to well-established guidelines among primary care physicians as well as specialists [10-12]. There are several ways to overcome this issue, including physician education [13], scheduled reminders, and recall systems [14-15]. An electronic health system is also an important tool that can be used to improve vaccination coverage by adding automatic reminders for patients with chronic liver diseases [16].

There are several limitations of this study. This was a retrospective study that was performed at a single center. We also did not record the severity of liver disease and assumed that the patient was not vaccinated if there were no documented vaccination on the chart. It is possible that those patients might have received vaccinations elsewhere.

The abstract of our research study was presented as a poster at the 2015 American College of Gastroenterology Annual Scientific Meeting by Iftikhar O, Mahmood S, Ali IA, et al.

## Conclusions

In summary, despite clear guidelines for the immunization of patients with chronic liver diseases, a significant number of patients are not adequately vaccinated against HAV and HBV, putting them at increased risk of contracting these infections. Educating resident physicians, developing vaccination protocols, and referral to the Gastroenterology clinic are likely to improve vaccination status for patients with chronic liver diseases.
